# Supplementary material for: Causal Relationship between Aging and Anorexia Nervosa: A White-Matter-Microstructure-Mediated Mendelian Randomization Analysis
Source: Biomedicines. 2024 Aug 16;12(8):1874. doi: 10.3390/biomedicines12081874 (PMC11351342; doi:10.3390/biomedicines12081874)
Supplement: Supplementary file 1 [file biomedicines-12-01874-s001.zip › Supplementary Figure/Figure S8/Leaveoneout plot of MR analysis for TL to ubm-b-1616.pdf]

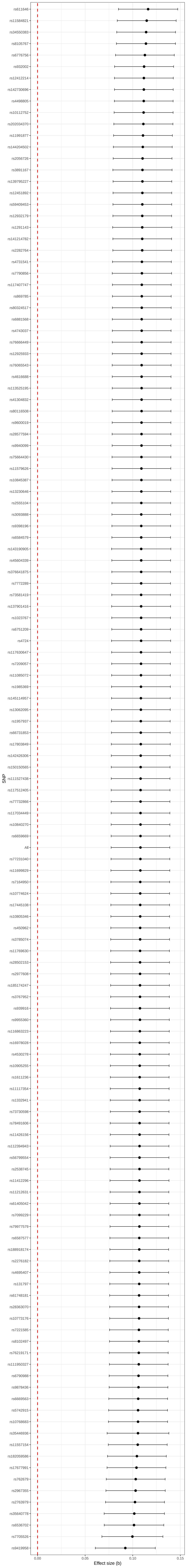
rs611646
rs11584821
rs34550383
rs8105767
rs6776756
rs932002
rs12412214
rs142730696
rs4498805
rs10112752
rs202034370
rs11991877
rs144204502
rs2056726
rs3891167
rs139795227
rs12451892
rs59409453
rs12932179
rs1291143
rs141214782
rs2282764
rs4731541
rs7790856
rs117407747
rs869785
rs80324517
rs6881568
rs4743037
rs76666449
rs12925933
rs76065543
rs4616688
rs113525195
rs41304832
rs80116508
rs9600019
rs28577594
rs9940099
rs75664430
rs11579626
rs10845387
rs13230646
rs2555104
rs3093888
rs9398196
rs6584579
rs143190905
rs45604339
rs376641875
rs7772289
rs73581419
rs137901416
rs1023767
rs6751209
rs4724
rs117630647
rs7209057
rs11085072
rs1985369
rs145114957
rs13062095
rs1957937
rs66731853
rs17803849
rs142426306
rs150150565
rs111527438
rs117512405
rs77732866
rs117034449
rs10840270
rs6659669
All
rs77231040
rs11699829
rs7164950
rs10774624
rs17445108
rs10805346
rs450962
rs3785074
rs11769630
rs28502153
rs2977608
rs185174247
rs3767952
rs939916
rs9955360
rs116863223
rs16978028
rs4530278
rs10905255
rs1611236
rs11117354
rs1332941
rs73730598
rs78491606
rs11426156
rs112394943
rs56799554
rs2538745
rs11412296
rs11212631
rs61405042
rs7099229
rs79977579
rs6587577
rs188918174
rs2276182
rs4695407
rs131797
rs61748181
rs28363070
rs10773176
rs7221585
rs8102497
rs76219171
rs111950327
rs6790988
rs9878436
rs6669563
rs5742915
rs10768683
rs35446936
rs11557154
rs182059586
rs17677991
rs762679
rs2967355
rs2763979
rs35640778
rs6536702
rs7705526
rs9419958
SNP
0.00
0.05
0.10
0.15
Effect size (b)
